# Supplementary figures and images for: Morphological characterization and sexual dimorphism of the antennal sensilla in Bactericera gobica Loginova (Hemiptera: Psyllidae)—a scanning and transmission electron microscopic study
Source: PeerJ. 2022 Feb 15;10:e12888. doi: 10.7717/peerj.12888 (PMC8855716; doi:10.7717/peerj.12888)

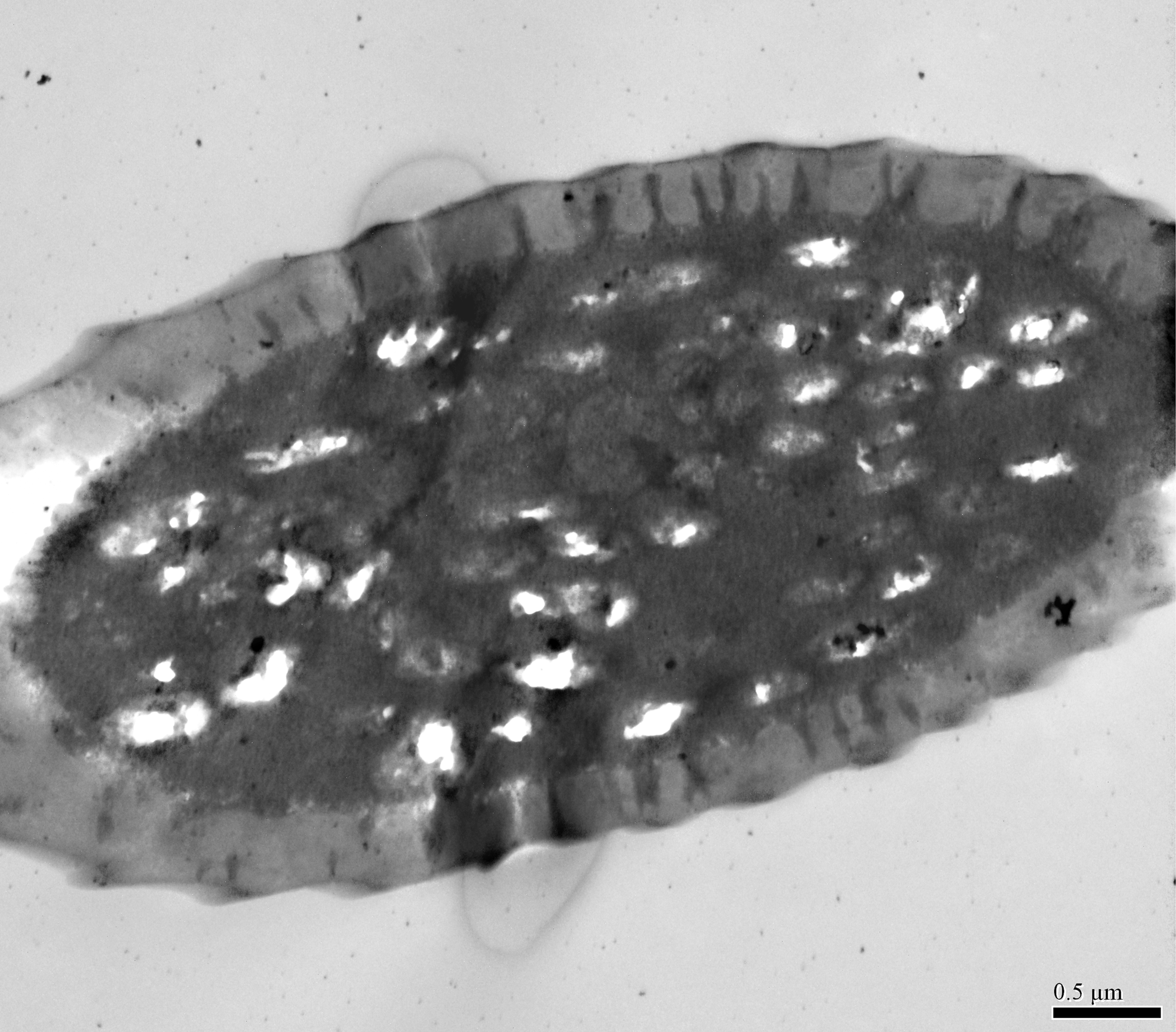

Supplement: Supplemental Information 2 [file peerj-10-12888-s002.png]

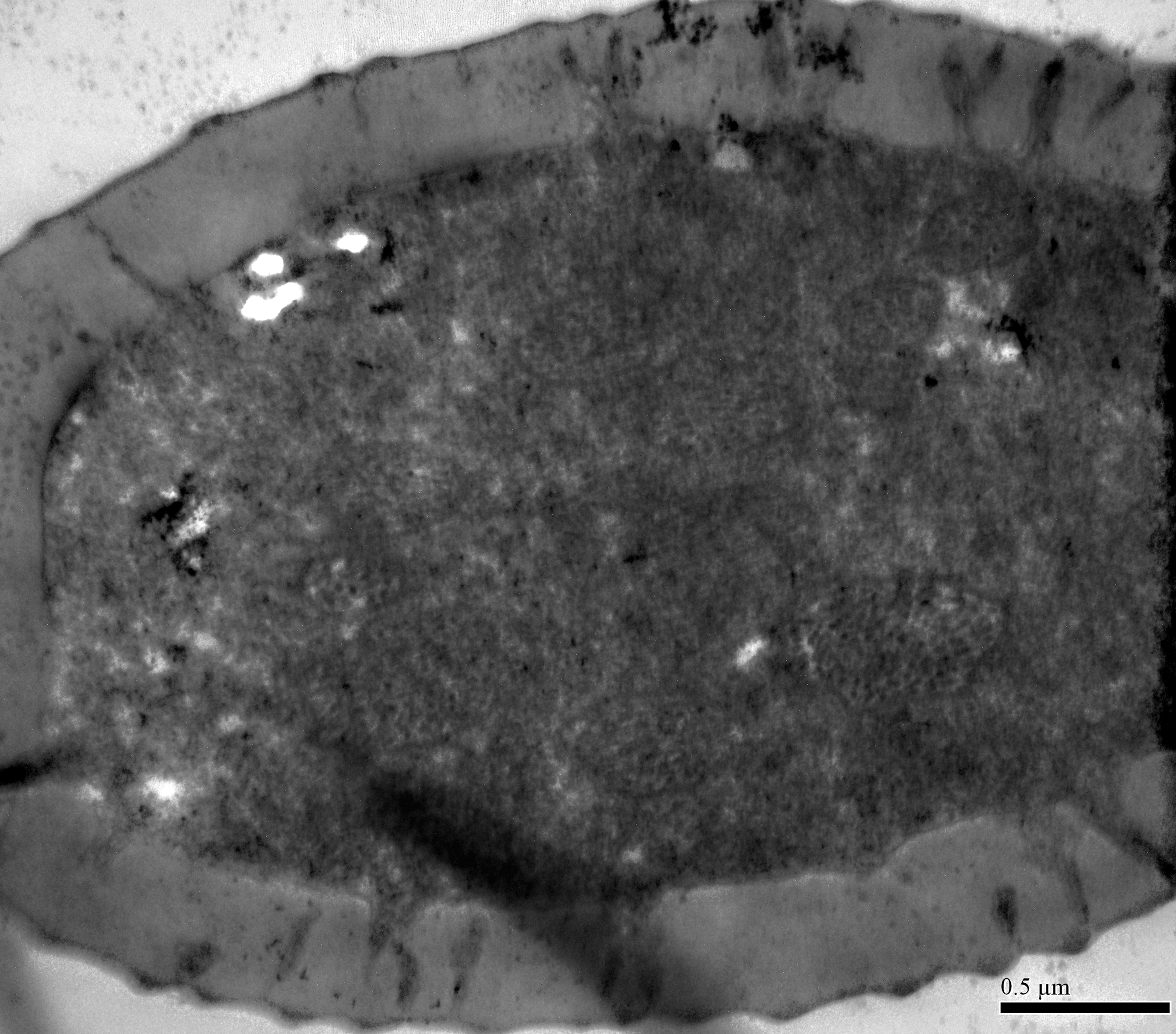

Supplement: Supplemental Information 3 [file peerj-10-12888-s003.png]

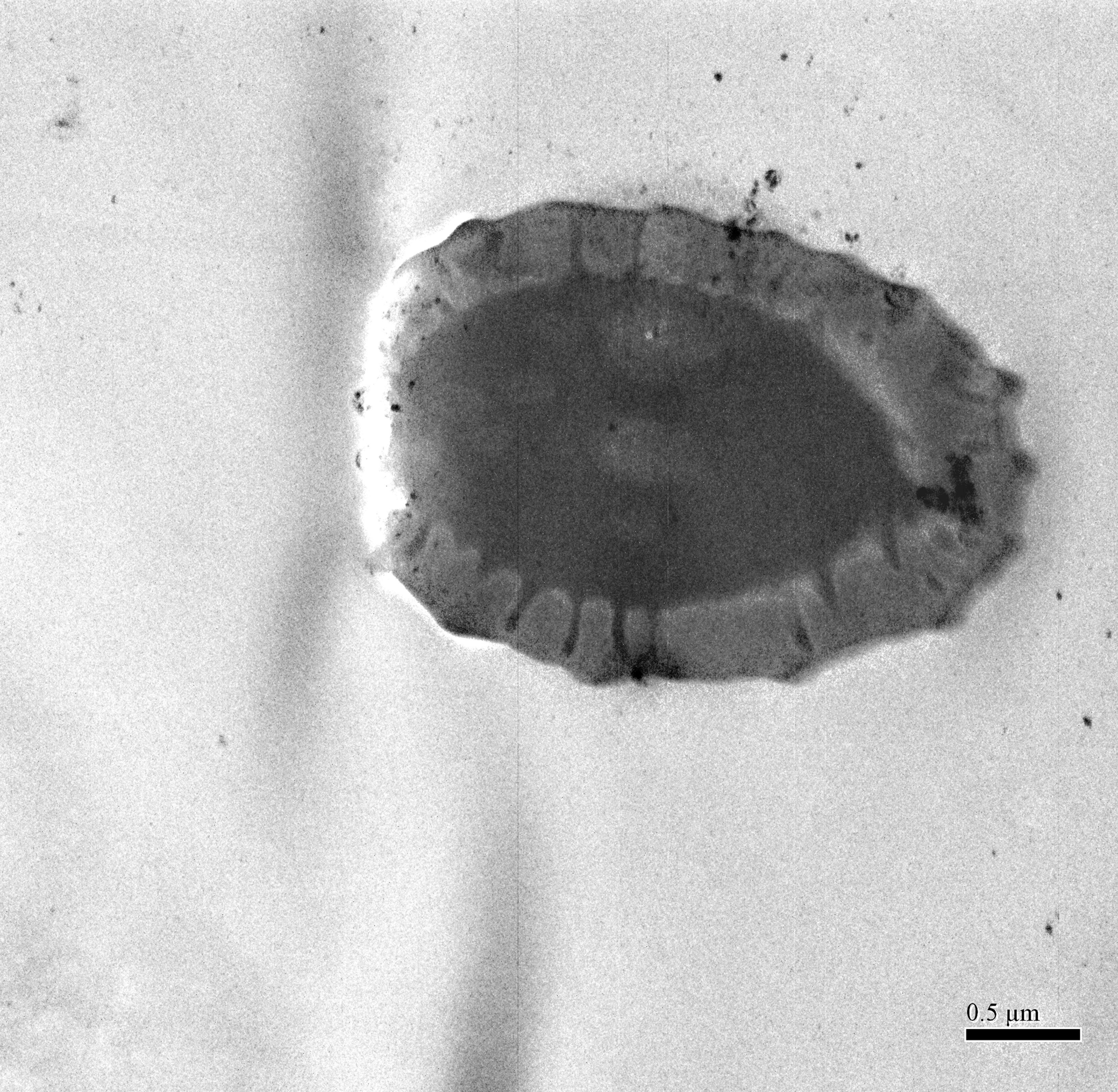

Supplement: Supplemental Information 4 [file peerj-10-12888-s004.png]

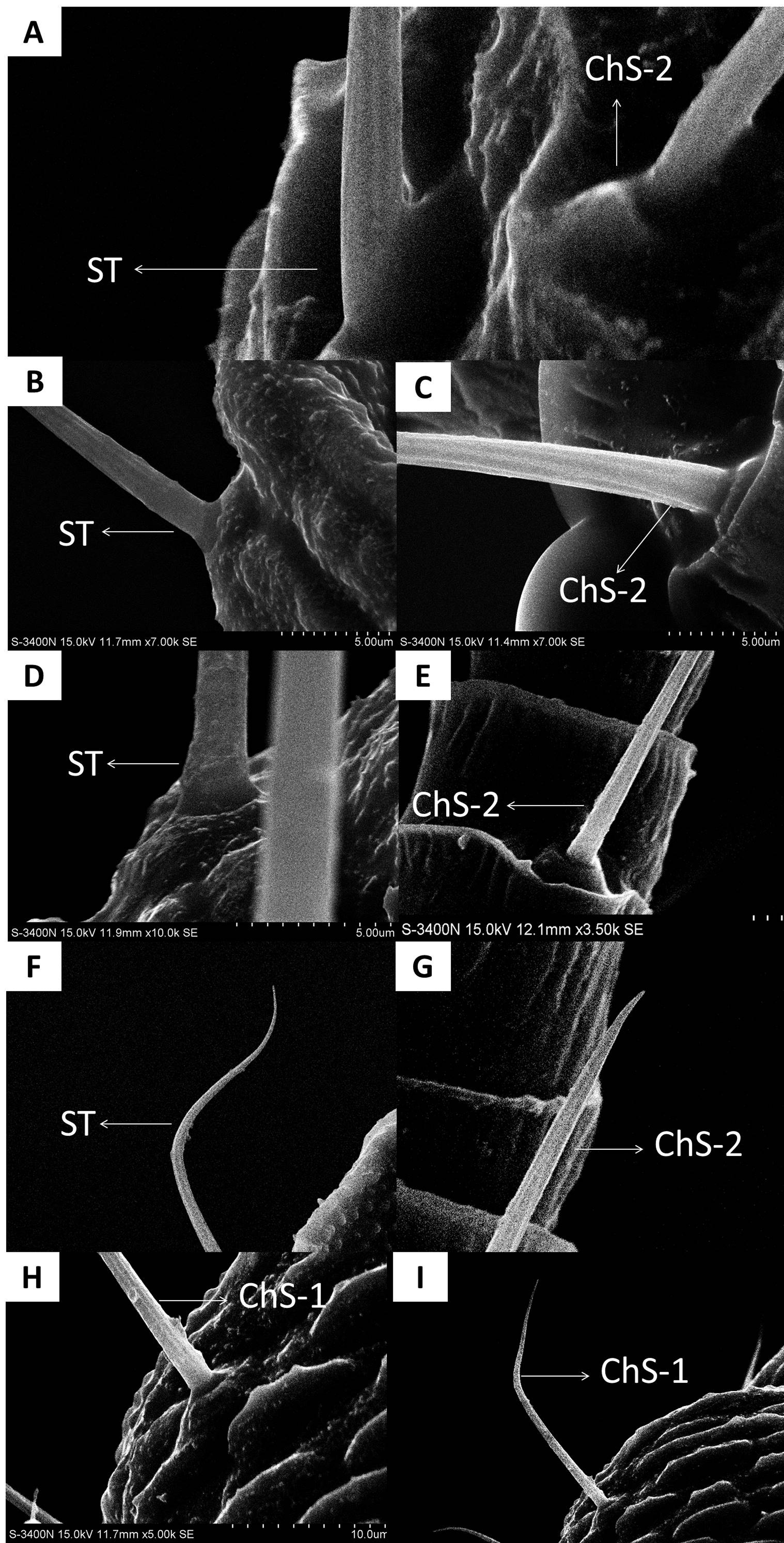

Supplement: Supplemental Information 5 [file peerj-10-12888-s005.png]
